# Crosstalk between decidual natural killer cells and extravillous trophoblasts at the maternal-fetal interface: current status and future perspectives

**DOI:** 10.3389/fimmu.2025.1703156

**Published:** 2025-10-22

**Authors:** Yeqi Qiu, Mingye Chen, Rong Lin, Xiaoyan Chen, Lichun He, Hui Yin, Xian Chen

**Affiliations:** ^1^ Department of Microbiology and Immunology, Guangdong Pharmaceutical University, Guangzhou, China; ^2^ Shenzhen Key Laboratory of Reproductive Immunology for Peri-implantation, Shenzhen Zhongshan Institute for Reproductive Medicine and Genetics, Shenzhen Zhongshan Obstetrics and Gynecology Hospital (formerly Shenzhen Zhongshan Urology Hospital), Shenzhen, China; ^3^ Guangdong Engineering Technology Research Center of Reproductive Immunology for Peri-Implantation, Shenzhen, China

**Keywords:** decidual NK cells, extravillous trophoblasts, maternal-fetal interface, pregnancy, invasion

## Abstract

The immune tolerance microenvironment is essential for the establishment and maintenance of pregnancy at the maternal-fetal interface. The maternal-fetal interface is a complex system containing various cells, including decidual stromal cells, lymphocytes, and trophoblasts. Decidual natural killer cells (dNKs) are the largest leukocytes and play a critical role in maintaining maternal-fetal immune tolerance and regulating the biological behaviors of extravillous trophoblasts (EVTs). Numerous studies have investigated the crosstalk between dNKs and EVTs at the maternal-fetal interface. On the one hand, dNKs can affect the invasion and migration of EVTs. On the other hand, EVTs can influence the immunological function of dNKs and the state of the maternal-fetal immune microenvironment. This review aims to summarize the most recent advancements in comprehending the phenotypes and functions of dNKs and EVTs, as well as their dynamic interactions that are crucial for the establishment and maintenance of pregnancy. Further developments in this area will greatly enhance both basic research and clinical applications in the field of reproductive medicine.

## Introduction

The success of a pregnancy relies on the complex interaction between maternal cells and the placental trophoblasts, which ultimately transform the uterus into a specialized environment capable of meeting the metabolic demands of a growing semi-allogeneic fetus while maintaining maternal tolerance (1). During the window of implantation (WOI), the human endometrium transforms into the decidua under the crucial influence of progesterone to support embryo implantation (2). The endometrial stromal, epithelial, endothelial, and immune cells coordinately create a receptive microenvironment in the uterus, and a synchronized embryo-endometrium crosstalk at this stage is a prerequisite for a successful implantation and pregnancy maintenance (3). Shortly after implantation, extravillous trophoblasts (EVTs) develop in placental anchoring villi and migrate into the maternal decidual stroma and vessels, differentiating into interstitial EVTs (iEVTs) and endovascular EVTs (eEVTs), respectively (4). The iEVTs eventually fuse to form placental bed giant cells (GCs) and migrate toward the maternal spiral arteries (SA), in combination with eEVTs, remodel them to become high-conductance vessels that can deliver a sufficient blood supply to the developing fetus (5). Numerous studies have contributed to current knowledge, stating that defects in this process can induce disordered blood flow into the intervillous space and damage the placental villous tree (6). Thus, abnormal placentation underlies adverse pregnancy outcomes, including recurrent miscarriage (RM), fetal growth restriction (FGR), pre-eclampsia (PE), and stillbirth (7–9). Despite these insights, the underlying molecular mechanisms regulating EVT development and function remain largely unexplored.

There have been extensive studies on the endometrial receptivity, particularly concerning the role of local immune cells in uterine hemostasis; however, recent interest has turned to the contribution of local immune cells at the maternal-fetal interface. Immune cells account for 40% of the human decidua (10). Uterine NK cells (uNKs) are found in the human endometrium and the decidua. They typically increase in number during the late secretory phase of the menstrual cycle and accumulate in the decidua before the appearance of fetal trophoblasts (11). The decidual NK cells (dNKs) account for about 70% of immune cells during the first trimester of pregnancy, with macrophages and T cells accounting for 10-30% (12, 13). After the first trimester, the number of dNKs will decline. dNKs have been detected throughout pregnancy in both the decidua basalis and parietalis, where trophoblasts are present and absent, respectively (11). As a result, it has been postulated that specific dNK subtypes play an important role in the development and function of EVTs. In this review, we examine our current understanding of how dNKs and EVTs interact to support the establishment and optimal development of the placenta. In particular, we highlight the recent findings that suggest that different functions of dNK cell subsets are crucial for controlling EVT fate. We discuss current evidence that the primary role of the EVTs is in shaping the receptive immunomodulatory environment of the maternal decidua, and we explain how the dNKs promote this. Thus, we focus exclusively here on the dialogue between the human dNKs and EVTs early in gestation. Finally, we discuss the outstanding questions in the field and make suggestions as to how these issues might be resolved.

## EVT differentiation and invasion during early pregnancy

The differentiation status of the trophoblast, from villous cytotrophoblasts (CTBs) into EVTs, involves a switch from a proliferative to an invasive, cytokine-secreting phenotype. Shortly after implantation, the trophectoderm (TE) that comprises the outer layer of the blastocyst transforms into mononuclear CTBs, which form placental villi through branching morphogenesis. CTBs further fuse into multinuclear syncytiotrophoblasts (STBs) in the floating villi and form the syncytial layer responsible for critical functions such as hormone production and clearance of fetal waste products (14). CTBs at branched anchoring villus tips have a proliferative phenotype and differentiate into EVTs. At the distal region of the cell column, EVTs invade the decidua up to the inner third of the myometrium (15). EVTs that migrate into the maternal decidua are called iEVTs and further develop into endovascular trophoblasts that migrate through the spiral arteries (16). EVT phenotypic change includes increased production of cytokines, proteases, and adhesion molecules, allowing them to migrate and invade into the uterine environment, and interact with different decidual cell subpopulations (17). Lack of decidua may lead to excessive EVT invasion in placenta accreta. In contrast, inadequate EVT invasion is linked to abnormal pregnancy complications such as RM and PE (18). A recent study has suggested that the abnormally low expression of MYB Proto-Oncogene Like 2 (MYBL2) in the placental trophoblasts may contribute to RM pathogenesis by disrupting EVT development and function (19).

It has been reported that cytokines, chemokines, and environmental oxygen can regulate the differentiation and invasion of EVTs. During the EVT invasion and interaction with the extracellular matrix (ECM), the expression of phenotypic adhesion molecules, such as integrin, vascular endothelial cadherin, platelet endothelial adhesion molecule, and vascular endothelial adhesion molecule, is upregulated (20–22), while the expression of E-cadherin and connexin-40 is downregulated (23, 24). In addition to changes in adhesion molecule expression, EVTs also upregulate a range of proteases, including matrix metalloproteinase (MMPs), cathepsins, and urokinase plasminogen activator (uPA) (25–27), to aid in their invasion of the decidua through the ECM. Furthermore, the role of decidual secreted factors in regulating EVT differentiation and invasion has been demonstrated. For instance, CCR1, which is expressed on human trophoblasts, interacts with its ligands CCL2 and CCL5, which are expressed by decidual cells, to promote EVT migration and initiate trophoblast invasion into the maternal tissue (28). However, some chemokines, such as CXCL6 and CXCL14, have been shown to inhibit trophoblast invasion by downregulating the expression of MMP-2 and MMP-9 (29, 30). Recent single-cell RNA sequencing (scRNA-seq) analysis has also identified the presence of programmed death-ligand 1 (PDL1) in EVTs and revealed new inhibitory interactions between killer cell lectin-like receptor B (KLRB1) and T cell immunoreceptor with Ig and ITIM domains (TIGIT) on dNK cells and C-type domain family 2 member D (CLEC2D) on EVTs (31). This research suggests that the damaging effects of maternal dNK cells on fetal EVTs can be circumvented in the microenvironment of the maternal-fetal interface. Therefore, the bidirectional communication between EVTs and dNKs emerges as a central regulator of EVT differentiation and invasion during pregnancy.

## Phenotypes and subsets of dNKs during pregnancy

dNKs are the most abundant immune cells at the maternal-fetal interface during early pregnancy. It is widely recognized that the phenotype and function of dNKs differ from those of peripheral NK cells (pNKs) (32). Phenotypically, CD56^bright^CD16^-^ NKs constitute the majority of human uNK cells, whereas CD56^dim^CD16^+^ NKs constitute the majority of pNKs (32, 33). Even though dNKs have a higher abundance of granulysin, granzyme A, and granzyme B, they are significantly less cytotoxic than pNKs. This could be because dNKs are unable to form mature activating synapses that contain granules (34). The main function of dNKs is to secrete cytokines and chemokines that promote angiogenesis and placentation. These functions are different from those of pNKs. It has been well known that dNKs highly express killer-immunoglobulin-like receptors (KIR2DL1, KIR2DL2/3, and KIR2DL4) and killer cell lectin-like receptors (NKG2E and NKG2A). These receptors can inhibit the cytotoxicity of dNK cells towards semi-allogeneic fetuses (35). In addition, dNKs express the activating receptors NKp46, NKp30, and NKp44. Evidence indicates that only the activation of NKp46 can induce dNKs degranulation and immune synapse formation (36), while the inhibitory form of NKp30 and NKp40 in dNKs can suppress the cytotoxicity with antibody stimulation (37).

**Figure 1:**
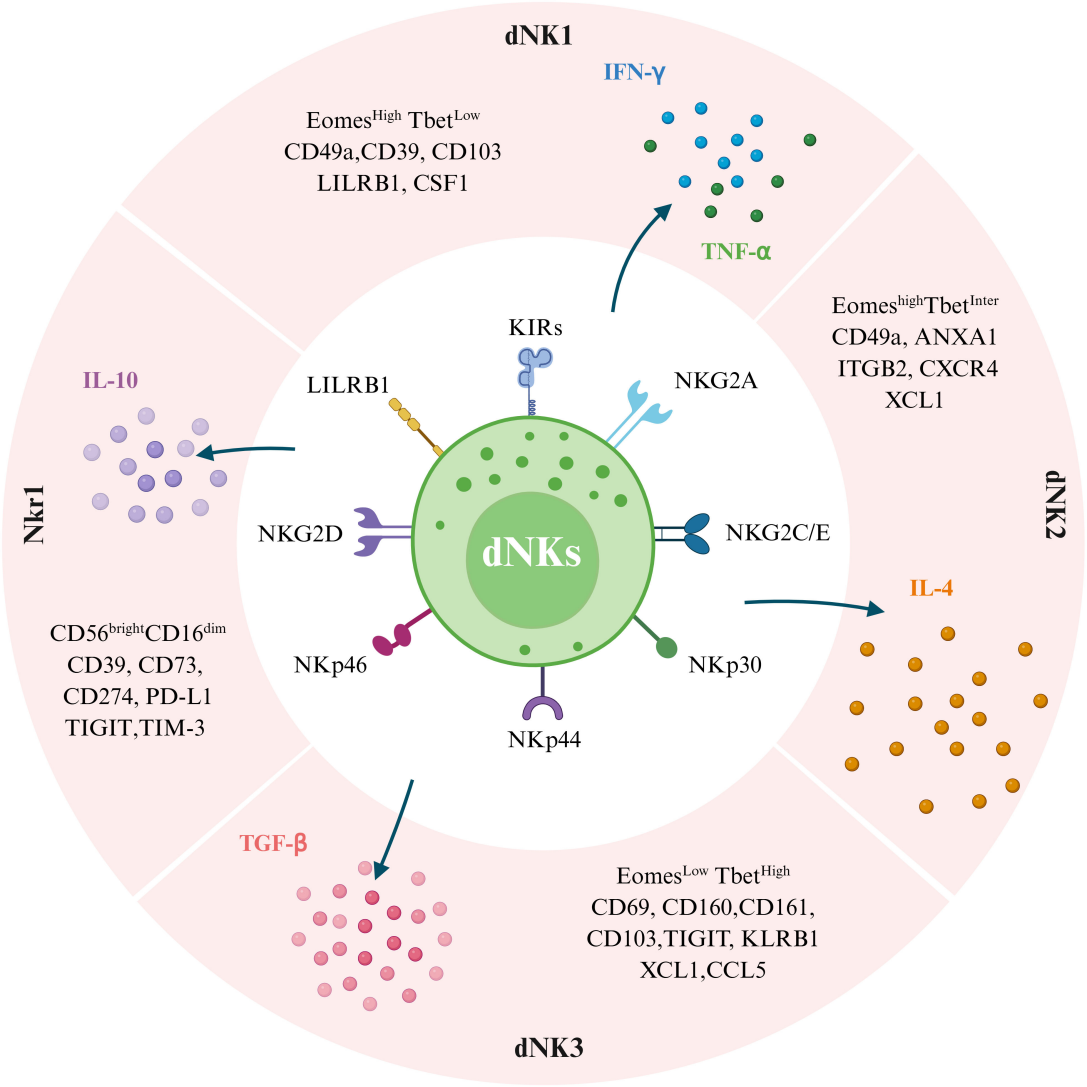
Subpopulations of human decidual natural killer cells (dNKs) at the maternal-fetal interface and their differences in surface marker expression profiles. Single-cell sequencing showed that dNKs were divided into four subpopulations, including dNK1, dNK2, dNK3, and NKr1. These cells have both inhibitory and activating receptors. Transcription factors (Eomes and Tbet) and cell surface markers (LILRB1, ITGB2, XCL1, CSF1, CXCR4, CD49a, CD160, and CD161), as defined by single-cell RNA sequencing and large-scale phenotyping, allow the distinction of dNK1, dNK2, and dNK3. NKr1 cells are CD56^bright^CD16^dim^ with high expression of CD39, CD73, CD274, PD-L1, TIGIT, and TIM-3 phenotype. Additionally, four dNK cell subtypes have different cytokine secretion capacities in response to nonspecific stimulation. Specifically, dNK1 cells predominantly secrete IFN-γ and TNF-α, dNK2 cells mainly produce IL-4, dNK3 cells are mainly secrete TGF-β, and NKr1 cells secrete IL-10. (Image created with BioRender.com, with permission).

Diverse subsets of dNKs are reported on different marker gene expressions in cells (**Figure 1**). Initially, much like the Th1/Th2/Th3/Tr1 (Type 1 T regulatory cells) paradigm, dNKs were categorized functionally into four groups based on their predominant cytokine profiles: NK1 (producing Th1-type cytokines), NK2 (producing Th2-type cytokines), NK3 (producing TGF-β), and regulatory NK1 cells (producing IL-10) (38, 39). Subsequently, with the development of scRNA-seq technology, new classifications have been developed based on surface marker expression have been established. Li et al. (40) identified four main subsets of NK cells in the endometrium at WOI, distinguished by the expression of CD49a and CXCR4: NK1 (CD49a^+^CXCR4^-^), NK2 (CD49a^+^CXCR4^+^), NK3 (CD49a^-^CXCR4^-^), and NK4 (CD49a^-^CXCR4^+^). These subsets showed dynamic and periodic changes throughout the menstrual cycle. Among them, the tissue-resident NK1 and NK2 subsets, which express CD49a (also known as ITGA1) and Eomes, were dominant in the endometrium during WOI and are considered potential progenitor cells of dNKs. Decidual CD49a^+^Eomes^+^NK cells have been reported to promote fetal growth during early pregnancy, and these cells in menstrual blood and decidua can reflect endometrial status and associate with RM (41). Building upon scRNA-seq data, several studies have reported slightly different classifications. Vento-Tormo et al. (31) redefined dNKs in the first-trimester decidua into three major dNK subsets (dNK1, dNK2, and dNK3), all of which express the tissue-resident markers CD49a and CD9. In contrast, Espino et al. (42) found that all three CD49a^+^dNK cell subsets (dNK1, dNK2, and dNK3) express the Emoes and Tbet transcription factors at varying levels, distinguishing them based on a gradient of Emoes^high^T-bet^low^ (dNK1) to Emoes^low^Tbet^high^ (dNK3), while dNK2 cells express intermediate levels of Emoes and Tbet. Together, the landscape of NK subsets is diverse and can be defined by different criteria, such as functional cytokine profiles or surface and transcriptional markers identified through high-resolution technologies.

## Allorecognition of trophoblast by dNKs

It is well known that dNKs guide trophoblast invasion through direct interaction between ligands and receptors or cytokine production (43). Killer-immunoglobulin-like receptors (KIRs) are paired receptors expressed on NK cells with both activating and inhibitory functions. Most inhibitory KIRs can recognize self-major histocompatibility (MHC) class I surface molecules and protect the target cells against the cytotoxic activity of NK cells, which is essential in facilitating self-tolerance (44). Human leukocyte antigen (HLA), also known as MHC, is the most polymorphic locus in the human genome, encoding the human MHC class Ia (HLA-A, HLA-B, and HLA-C), class Ib (HLA-E, HLA-F, HLA-G, and HLA-H), and class II (HLA-DR, HLA-DQ, HLA-DM, HLA-DO, and HLA-DP) molecules (45). There is general agreement about the expression of HLA molecules by trophoblast (46). EVT is in direct contact with all maternal decidual cells. To prevent maternal immune rejection, EVTs express non-classical HLA-G, HLA-E, and polymorphic HLA-C molecules, but not the typical class I HLA-A or HLA-B molecules during pregnancy. HLA-G exclusively expressed in EVTs, is a crucial factor in establishing maternal-fetal immune tolerance by interacting with inhibitory KIRs on dNKs. Recently, Gu et al. (47) conducted a genome-wide CRISPR-Cas9 screen and discovered that the WNT pathway has a negative effect on HLA-G. They also found that the transcription factors TEAD1 and TEAD3 are crucial for HLA-G transcription, which provides insights into how HLA-G expression can be controlled in EVTs to protect allogeneic cells from immune rejection. Among EVT-expressed HLA molecules, the function of HLA-C and HLA-G has been studied extensively, but relatively little is known about HLA-E and HLA-F. A recent study in patients with PE has shown that HLA-F in HLA-G^+^EVTs was significantly downregulated in PE samples compared to controls (48). Thus, it is believed that, since HLA-F and HLA-G share certain expression quantitative trait loci in common, these two genes may act in concert in EVTs.

The effector function of NK cells is dependent on a balance between the signals received by activating and inhibitory receptors (49). Generally, the functional inhibition of NK cells is mediated either by CD94-NKG2A binding to HLA-E or by inhibitory members of the diverse KIR family binding to HLA class I molecules (50). Multiple functional and genetic studies have provided evidence that dNKs can recognize and respond to EVT through KIR-HLA interactions. dNK1 cells express higher levels of inhibitory receptors (KIR2DL1, KIR2DL2, KIR2DL3) and activating receptors (KIR2DS1 and KIR2DS4) with high affinity for HLA-C molecules, as well as LILRB1 with high affinity for binding to HLA-G molecules expressed on EVT (31). Both dNK1 and dNK2 express activating NKG2C and NKG2E, and inhibitory NKG2A receptors for HLA-E molecules, suggesting that dNK1 cells play a critical role in the recognition and response to EVTs (31). In comparison to dNK1 cells, other evidence suggests that dNK2 and dNK3 cells secrete more XCL1 chemokines, which have interactive receptors on both maternal dendritic cells (DCs) and EVTs (51). In addition, dNK3 cells express high levels of CCL5, which is the ligand of CCR1 expressed on EVTs, indicating that dNK3 cells may also play an important role in regulating EVT invasion (28). Overall, these findings suggest that dNKs play a key role in interacting with EVTs and responding to EVTs.

## The crosstalk between dNKs and EVTs at the maternal-fetal interface

### How do dNKs affect EVTs?

Precise regulation of the proliferation, apoptosis, differentiation, migration, and invasion of EVTs is essential for the establishment and maintenance of pregnancy. Insufficient EVTs invasion could result in RM, PE, and restrict fetal growth, while excessive EVTs invasion could lead to placental accreta and maternal postpartum hemorrhage (52). In the local microenvironment, dNKs are typically located near trophoblasts and are usually thought to regulate EVT invasion. Hence, the paracrine activity of dNKs exerts an important role in the biological function of EVTs. To better understand the role of dNKs in EVT biological behavior, various *in vitro* experiments have been employed, seeming to depend on the particular invasion assays and trophoblast cells that are used (53).

In a spheroid invasion assay, the additional conditional medium from dNKs obtained during early pregnancy could significantly increase the invasion of the EVT cell line and induce the outgrowth of EVT explants from villous tissue (54). dNKs could express and secrete interleukin (IL)-8, interferon-inducible protein (IP)-10, vascular endothelial growth factor (VEGF), and placental growth factor (PLGF). Both *in vitro* and *in vivo* assays have demonstrated that neutralizing antibodies against IL-8 and IP-10 can partially inhibit the migration of HLA-G^+^ trophoblasts toward dNKs (55). Moreover, it has been found that HLA-G can bind to KIR2DL4 on dNKs, leading to the activation of IL-8 production (56, 57), suggesting that human dNKs play a role in promoting EVT invasion by secreting IL-8 and IP-10.

dNKs were also found to express and secrete interferon (IFN)-γ and tumor necrosis factor (TNF)-α, which inhibited EVT invasion by increasing cell apoptosis (58, 59). The invasive capacity of villous explant-derived EVTs was found to decrease when treated with 10 ng/mL IFN-γ. However, this effect was reversed when neutralizing antibodies against IFN-γ were added, with IFN-γ inhibiting EVT invasion by inducing their apoptosis (59). Moreover, dNKs obtained during the first trimester inhibited the outward migration of EVTs from the column edge of villus explants. This inhibition was blocked by a neutralizing antibody against IFN-γ. The inhibition specifically targeted EVT migration and was associated with an increase in MMP-2 and MMP-9 activity and a decrease in plasminogen activator inhibitor-1 (PAI-1) levels (60, 61), suggesting a direct role for dNKs in modulating EVT differentiation as they form columns and then migrate from anchoring villi.

Besides, a recent study utilizing trophoblast organoid models to simulate interactions between dNKs and EVTs found that dNK-derived factors, such as colony-stimulating factor (CSF)-1, CSF2, XCL1, and CCL5, bind to specific receptors on EVTs (62). This binding not only influences EVT development and differentiation, but also influences angiogenesis and nutrient supply. In parallel, there has also been a study highlighting the promoting effect of macrophage colony-stimulating factor (M-CSF)/M-CSFR signaling on EVT proliferation and differentiation (63). *In vivo* data from humanized NOG mice and the *in vitro* results from trophoblast stem cells collectively elucidate the pivotal role of human CD56^+^CD39^+^ dNKs in controlling trophoblast cell differentiation fate, encompassing both invasive and syncytial pathways by secreting M-CSF (64). These findings suggest that dNK-produced M-CSF may act as a crucial factor to regulate diverse pathways involved in EVT differentiation to maintain placental health and fetal well-being. Recent scRNA-seq analysis provides insights into the potential EVT1 and dNK1/2 interaction mediates the chemotaxis of EVT1 and facilitates the regulation of endothelial cell death, initiating spiral artery remodeling. The loss of this specific cellular interaction may result in RM (65). Altogether, there are multiple mechanisms in the decidua to dampen potentially damaging dNK responses to EVT.

**Figure 2:**
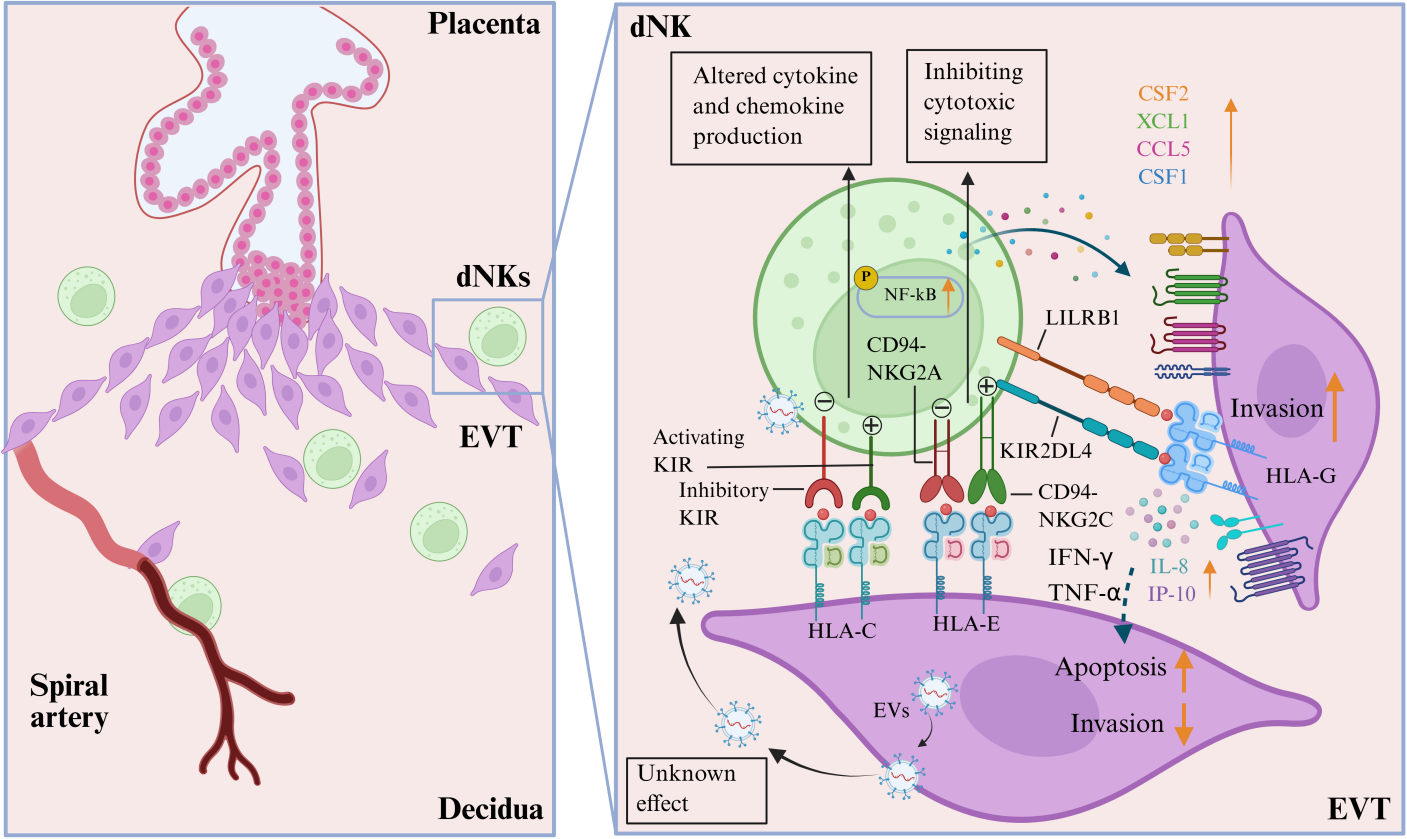
Interactions of decidual natural killer cells (dNKs) and extravillous trophoblast (EVTs) at the maternal-fetal interface. Different combinations of the highly polymorphic maternal killer cell immunoglobulin-like receptors (both activating and inhibitory KIRs) expressed by the dNKs and fetal HLA-C/G/E expressed by EVTs can lead to altered secretion of cytokines and chemokines by dNKs (CSF2, XCL1, CCL5, and CSF1), which are associated with the development of placentation. The effect of HLA-E expressed by EVT engaging inhibitory CD94-NKG2A is to inhibit cytotoxic signaling, while the role of activating CD94-NKG2C receptors on dNKs is not yet fully understood. LILRB1 has a high affinity for binding to HLA-G molecules expressed on EVTs. The interaction between HLA-G on EVTs and KIR2DL4 on dNKs activates the NF-κB signaling pathway in dNKs, promoting the secretion of interlukine-8 (IL-8) and interferon-inducible protein-10 (IP-10), which in turn promotes EVT invasion. However, the secretion of IFN-γ and TNF-α secreted by dNKs can also lead to the apoptosis of EVTs and inhibit their invasion. Additionally, EVTs can also regulate the immune function of dNKs by secreting extracellular vesicles (EVs), although the exact mechanisms of this action are still unknown. (Image created with BioRender.com, with permission).

In summary, the dualistic effects of dNKs on EVT invasion are not arbitrary, but rather determined by specific receptor-ligand interactions, the predominance of distinct dNK subsets, and the local cytokine milieu (**Figure 2**). This functional switch can be seen as a balance between pro-invasive and anti-invasive signaling networks. The pro-invasive signaling is activated by engaging activating receptor-ligand pairs, such as KIR2DS1/HLA-C2 and HLA-G/KIR2DL4, which stimulate dNKs (particularly dNK1 and dNK2 subsets) to secrete a combination of pro-invasive factors, including IL-8, IP-10, M-CSF, and CSF1/2. This environment promotes EVT migration and vascular remodeling. On the other hand, the anti-invasive signaling is characterized by signaling through inhibitory receptors like NKG2A/CD94 upon binding HLA-E. This is further enhanced by the secretion of IFN-γ and TNF-α. These cytokines can induce EVT apoptosis and alter protease activity, thereby limiting invasion. Therefore, the overall effect on EVTs is likely a context-dependent integration of these competing signals. A change in any component, such as a different HLA-C allotype, polarization of dNK subsets, or varying levels of IL-8 versus IFN-γ, can shift the balance and impact the success of invasion, ultimately affecting pregnancy outcomes.

## The effect of EVTs on dNK cell functions

From the other way around, dNK functions are influenced by the surrounding environment and factors in contact with them. These factors include cytokines, chemokines, growth factors, etc. Among these, cell-cell interaction plays a key role. At the maternal-fetal interface, dNKs and EVTs are accumulated, and trophoblasts secrete factors that are essential in regulating the differentiation and function of dNKs. However, the specific factors involved in this process have not yet been fully understood. In this section, we will primarily focus on the impact of EVTs on dNK functions.

The classical pathway by which EVT regulates dNK function is via receptor-ligand interactions during pregnancy. Distinct HLA molecules expressed on EVT mediate targeted immunomodulatory effects by binding to specific receptors on dNKs (66). For example, HLA-E on EVT binds to NKG2A/CD94 on dNKs, thereby inhibiting cytotoxic signaling. The non-classical HLA-G molecule on EVTs recognizes ILT2 and KIR2DL4 on dNKs can induce various cytokine secretion and facilitate immune tolerance at the maternal-fetal interface. It has also been reported that the interaction between HLA-G and KIR2DL4 can activate the NF-κB signaling pathway, leading to the reprogramming of dNKs into a senescence-associated secretory phenotype. This, in turn, promotes the secretion of IL-6, IL-8, and IFN-γ, which facilitates EVT invasion and angiogenesis (67). NF-κB regulates the expression of these cytokines and influences NK cell phenotype and function, further promoting the crosstalk between EVTs and dNKs, ultimately contributing to normal spiral artery remodeling (68). Moreover, evidence from multiple functional and genetic studies supports the view that dNKs can recognize and respond to EVT via the interaction of KIR and HLA-C. Specifically, the interaction between the KIR2DS1 receptor and HLA-C2 stimulates dNKs to secrete XCL1 and CSF2, promoting EVT invasion and providing protection against PE (62). Research has demonstrated that the risk of PE is increased when the mother lacks the KIR2DS1 receptor and the fetus carries the HLA-C2 genotype (69). Additionally, studies have found that the expression of KIR2DS5 in the decidua of women with PE is significantly lower compared to healthy pregnancies. This decrease in KIR2DS5 activates the JAK2/STAT5 pathway, which suppresses the secretion of CSF2 by dNKs, ultimately affecting trophoblast function (70). In a recent scRNA-seq study, a novel interaction between FAM3C and HLA-C in dNK-EVT crosstalk was discovered under an organoid co-culture condition (71). FAM3C is a cytokine triggering the epithelial-mesenchymal transition (EMT) program and regulates various proteins, including Ras, STAT3, TGF-β, and LIFR, providing valuable resources for further application on the mechanism of EVT differentiation and interactions with dNKs.

EVTs have also been reported to modulate the immune properties of dNKs by regulating their metabolism and migration during placental development. EVTs can recruit dNKs to the decidua by secreting CCL2, and then facilitate angiogenesis and modulate the immune microenvironment during pregnancy (72). Conversely, EVTs carrying the STOX1 Y153H mutation exhibit significantly reduced levels of IL-6, IL-8, CCL2, and CXCL1, which impairs dNKs recruitment and migration and ultimately decreases their involvement in spiral artery remodeling in early-onset PE (73). In addition to soluble factors, extracellular vesicles (EVs) have also been shown to be another crucial medium in the communication network at the maternal-fetal interface in recent years (74). In fact, little is known about trophoblast-derived extracellular vesicles-mediated crosstalk between dNKs and EVTs up to now. To date, only one publication has reported that EVTs secrete trophoblast-derived extracellular vesicles that stimulate glycolysis and oxidative phosphorylation in dNKs via HLA-E. This metabolic shift promotes dNKs to secrete VEGF and IFN-γ, both of which are crucial for spiral artery remodeling (75). Therefore, more evidence is needed to confirm the role of EVs in the regulation of dNK functions during pregnancy.

Although numerous studies in recent years have focused on the regulation of EVT development and differentiation by NK cells, there are still several unresolved scientific questions that require further investigation. One fundamental unanswered question is whether dNKs improve or inhibit trophoblast invasion. While a successful pregnancy requires EVTs to transform the maternal arteries, this process must also be carefully regulated to prevent excessive invasion. Unfortunately, most trophoblast invasion assays have not taken into account the potential functional effects of dNKs, as determined by genetic studies on maternal KIR-fetal HLA-C combinations (76). Another important issue in attempting to elucidate is how the phenotype and functions of EVTs change as they invade deeper into the decidua, or how EVTs stop invasion and fuse to become placental GCs in the myometrium, particularly under the influence of dNKs. To study this will require samples taken from pregnant hysterectomies, which is a limited source due to the rare procedure in early gestation. Experiments are required that reflect the normal *in utero* environment and can systematically determine how responses generated by specific dNK cell receptors regulate EVTs.

## The way forward: research priorities and clinical translational potential

Over the past two decades, research has established a consensus on the importance of balanced EVT differentiation and invasion during human placental development, the unique profile of dNKs, the expression of HLA molecules by EVT subpopulations, and the dNK-mediated allorecognition system dependent on KIR-HLA molecular interactions. It has also highlighted the need for immune tolerance to the trophoblast. However, as discussed, many questions remain about the dialogue between the dNKs and EVTs in human pregnancy. These questions have been historically difficult to address due to ethical and practical issues surrounding access to human tissues and the limitations of animal models.

In order to advance our understanding of crosstalk between dNKs and EVTs in human placentation success, it will be essential to apply new research technologies. These may include scRNA-seq and single-nucleus RNA sequencing, which enable the identification of novel cell populations, genetic markers, and potential functional pathways for further validation (77–79). Additionally, the use of single-cell spatial transcriptomics methods and multiplex antibody-based imaging can help visualize the dynamic changes occurring in the placenta during early pregnancy (80). However, it is important to note that there are considerable differences between human and mouse placentation, and while placentation in higher primates more closely resembles that of humans, interstitial EVT invasion is only observed in great apes (81). Therefore, no animal model is perfect for studying the interaction between dNKs and EVTs.

Experimenting with human trophoblast cells *in vitro* has posed challenges for researchers due to the limited relevance of available cell lines to EVTs *in vivo*. Previous studies have utilized choriocarcinoma-derived cell lines, such as HTR-8/SVneo and Bewo, which do not accurately represent the characteristics of normal invasive EVTs. In contrast, human trophoblast stem cell lines and trophoblast organoids can be induced to differentiate to invasive EVTs, offering more physiologically relevant models to investigate dNK-mediated regulation of EVT biological behavior. Despite much evidence to suggest that dNKs regulate EVT invasion, their precise functions still remain largely unclear. The necessity to use primary dNKs from ongoing early pregnancies to study these functions has obvious ethical and logistical limitations, and no representative dNK cell lines exist. Although genetic studies have linked specific combinations of maternal KIR and fetal HLA-C variants to an increased risk of PE and other pregnancy complications, the exact functional mechanisms are still unresolved. The use of novel EVT models, created from trophoblast stem cell lines and trophoblast organoids, which can be biobanked and HLA-C typed, presents a promising opportunity to explore the functional interaction between KIR-expressing dNKs and EVTs (44). The glycosylation of trophoblast proteins should also be a focus of future research into the crosstalk between dNKs and EVTs.

In spite of this, EVs, as an emerging intercellular communication medium, have been gradually proven to exert a role in mediating the mutual communication between cells. However, the role and potential mechanisms of EVs in the crosstalk between dNKs and EVTs at the maternal-fetal interface are rarely investigated, which is an emerging field that needs to be explored urgently. Moreover, in addition to dNKs, there are other immune cells present at the maternal-fetal interface, including macrophages, T cells, and dendritic cells. These cells also play important roles in regulating the biological behavior of EVT. It is worth investigating whether other cells are involved in the interaction between dNKs and EVTs, potentially establishing three-cell or multi-cell communication and contributing to the establishment and maintenance of pregnancy. This is an area that should be explored in future research.

The increasing understanding of the interaction between dNKs and EVTs presents promising opportunities for clinical application. These interactions can be targeted for the development of new diagnostic and therapeutic approaches for pregnancy disorders caused by impaired placental function. One potential application is the use of the molecular signature of dNK-EVT dialogue as a source of biomarkers. Specific combinations of KIR and HLA-C, as well as cytokine profiles and EVT-specific molecules, could be developed into prognostic panels (82, 83). These markers could be analyzed in maternal blood or cervical fluid to identify women at high risk for conditions such as RM, PE, or FGR, allowing for personalized monitoring and early intervention. Another potential application is the use of immunomodulatory therapies to target the dNK-EVT axis. For example, in cases of inadequate placental invasion, therapies could be designed to enhance the pro-invasive and pro-angiogenic functions of dNKs, potentially using low-dose cytokines or growth factors (84). In hyperinflammatory conditions, biologics that block specific activating KIRs or supplement tolerogenic signals could help restore immune balance. Additionally, the development of dNK cell-based therapies, where dNKs are expanded *in vitro* and then reinfused to support placental development (85), could be a promising future direction. The study of dNK-EVT crosstalk also has potential implications for ART. By assessing the uterine immune environment, including the phenotype and receptivity of dNK precursors, it may be possible to evaluate endometrial receptivity in IVF cycles. Furthermore, understanding how parental KIR and HLA-C genotypes interact could improve risk stratification for couples undergoing ART, potentially guiding clinical decisions such as single embryo transfer to optimize pregnancy outcomes. By bridging deep mechanistic research with these clinical applications, the study of dNK-EVT crosstalk has the potential to make tangible contributions to improving maternal and fetal health worldwide.

## Conclusions

The crosstalk between dNKs and EVTs is crucial for regulating trophoblast invasion, promoting spiral artery remodeling, and maintaining immune tolerance at the maternal-fetal interface. In this review, we have summarized recent advancements in understanding the context-dependent functional characteristics of dNKs and EVTs. Changes in the quantity, phenotype, and function of dNKs have been closely linked to pregnancy complications such as RM, PE, and FGR. These changes can result in restricted EVT differentiation and invasion, impaired spiral artery remodeling, and disrupted immune tolerance, all of which contribute to the development of pregnancy disorders. The decidual microenvironment and interactions between dNKs and other cells at the maternal-fetal interface, particularly their reciprocal regulation with EVTs, significantly impact the function of dNKs. Therefore, understanding the interaction between dNKs and EVTs is essential for elucidating the pathogenesis of pregnancy disorders. Future research focusing on the molecular mechanisms that regulate the biological function of dNKs and EVTs may provide new strategies for early diagnosis and intervention of pregnancy failure.
